# Employment of the Ascending Aortic Volume as a Predictor of Adverse Outcomes in Patients With Bicuspid Aortic Valve Disease

**DOI:** 10.31083/RCM42793

**Published:** 2026-02-06

**Authors:** Haowei Li, Yuekang Hu, Haiyun Yuan, Tianyu Chen, Jian Zhuang

**Affiliations:** ^1^Department of Cardiovascular Surgery, Guangdong Cardiovascular Institute, Guangdong Provincial People’s Hospital, Guangdong Academy of Medical Sciences, 510080 Guangzhou, Guangdong, China; ^2^Department of Cardiovascular Surgery, Sun Yat-sen Memorial Hospital, Sun Yat-sen University, 510080 Guangzhou, Guangdong, China

**Keywords:** bicuspid aortic valve (BAV), volume–height index (VHI), ascending aortic volume, computed tomography angiography (CTA)

## Abstract

**Background:**

A bicuspid aortic valve (BAV) is a common congenital heart disease. The primary treatment for this condition involves the surgical replacement of both the aortic valve and the ascending aorta, typically through the Bentall procedure. Traditionally, the timing of surgery in patients with BAV and aortic dilation is based on the maximum ascending aortic diameter. However, numerous patients who experienced adverse outcomes did not fulfil the established surgical criteria, highlighting the necessity for new predictive factors to guide surgical decisions more effectively. Thus, this study aimed to identify alternative parameters in patients with BAV that could serve as early indicators of surgical intervention and to establish clear threshold values.

**Methods:**

A retrospective analysis was conducted among 101 patients diagnosed with BAV at our institution between January 2004 and December 2023 who underwent follow-up computed tomography angiography. Demographic and clinical data were collected, focusing on the influence of ascending aortic volume on adverse outcomes, measured from the aortic annulus to the origin of the brachiocephalic artery.

**Results:**

The average ascending aortic volume, length, and diameter were 99,496.51 mm^3^, 90.94 mm, and 38.79 mm, respectively. Logistic regression analysis identified that only ascending aortic volume (*p* = 0.0338) and volume-to-height ratio (*p* = 0.0331) were significantly associated with adverse outcomes. In a multiple logistic regression model, the volume–height index (VHI) was independently associated with adverse outcomes (odds ratio (OR) 1.0008, 95% confidence interval (CI) 1.00023–1.00182; *p* = 0.048). Receiver operating characteristic (ROC) analysis determined the optimal cutoff value for the VHI as 66,340.5 mm^3^/m (area under the curve (AUC) = 0.797, 95% CI 0.676–0.896). The Kaplan–Meier curve showed that the event-free survival rate of patients with a VHI >66,340.5 mm^3^/m was consistently lower than that of the low VHI group; The difference between the two groups was statistically significant (log rank *p* < 0.0001).

**Conclusion:**

The VHI is a strong predictor of adverse outcomes in patients with a BAV and can guide surgical intervention decisions.

## 1. Introduction

Bicuspid aortic valve (BAV) is a congenital heart disease that is characterized by an aortic valve with two cusps instead of three. It is the most commonly occurring congenital heart condition in adults, which affects 0.5% to 2% of the general population [1, 2]. BAV is associated with a higher mortality rate and an increased risk of complications such as aortic regurgitation, stenosis, dilation, and dissection compared to other congenital heart defects [3]. Approximately 35% of the patients with BAV develop complications involving the aortic valve or ascending aorta, with most requiring surgical intervention [4]. The surgical decisions are primarily based on the diameter of the ascending aorta in patients with aortic dilation. Since 1998, the threshold for surgical intervention has been adjusted multiple times. According to the 2014 American College of Cardiology (ACC)/American Heart Association (AHA) guidelines [5], the size of the aortic root or ascending aorta should be monitored. The repair of the aortic sinus or replacement of the ascending aorta may be necessary when the diameter of the aortic sinus or ascending aorta exceeds 5.5 cm. The repair of the aortic sinus and replacement of the ascending aorta are considered reasonable if the diameter of both the aortic sinus and ascending aorta is greater than 5.0 cm, and the patient has risk factors for aortic dissection, such as a family history of aortic dissection or a diameter increase of 0.5 cm or more annually. Moreover, replacement of the ascending aorta is considered appropriate if the diameter of the ascending aorta is greater than 4.5 cm, and the patient undergoes aortic valve surgery because of severe aortic stenosis or regurgitation [6]. This discrepancy highlights the necessity of identifying other predictive factors, more accurately guiding surgical decisions, and optimizing the timing of interventions [7]. A strong association between aortic dilation in patients with BAV and a higher risk of severe aortic events, including aneurysms, dissection, and rupture, is revealed through review of the literature. Recent research also suggests that the length of the ascending aorta plays a significant role in the prediction of the formation of aneurysms [8]. The volume of the ascending aorta can be considered as an indicator of surgical intervention in patients with ascending aortic aneurysm, as it demonstrates a stronger association with pathogenic hemodynamic conditions [9]. Meanwhile, measurements of ascending aortic volume demonstrate higher diagnostic accuracy for acute type A aortic dissections (ATAADs) compared to the maximum diameter, enabling timely identification of the patients at risk [10]. Based on these findings, this study aims to explore the potential of ascending aortic volume as a combined measure of the diameter and length of the aorta, and to determine its ability to predict adverse outcomes in patients with BAV.

## 2. Methods

### 2.1 Study Population

This retrospective cohort study was performed at the Cardiovascular Disease Center in China. The ethics committee of the Cardiovascular Disease Center in China provided ethical approval for the study, which was conducted in accordance with the China Good Clinical Practice in Research guidelines. This study was conducted in accordance with the Declaration of Helsinki. Although current guidelines for anonymous retrospective studies may grant a waiver for informed consent, written informed consent was obtained from all participants in this study. [11]. A retrospective analysis of 101 patients diagnosed with BAV was performed at our institution between January 2004 and December 2023. Inclusion criteria were as follows: (1) Patients with complete computed tomography angiography (CTA) images; (2) those with complete clinical data; and (3) those with no severe mental illness or systemic disease affecting the assessment. The following were the exclusion criteria: (1) patients without accessible or high-quality CTA scans; (2) those with missing demographic data; (3) those with other congenital aortic abnormalities; and (4) those diagnosed with Marfan syndrome.

### 2.2 Data Collection and Definition

Baseline clinical characteristics, comorbidities, and imaging data were retrieved from the electronic medical records. The occurrence of adverse outcomes was considered the primary endpoint, which was further defined as the development of an aortic aneurysm, aortic dissection, or death. An aortic aneurysm, according to the 2022 ACC/AHA guidelines, is defined as an aortic diameter of ≥5.0 cm or 1.5 times the normal diameter (typically 2.1–3.4 cm, which is adjusted for age, sex, and body surface area) [12]. These events were further confirmed using at least one of the following sources: autopsy reports, surgical records, death certificates, or radiological imaging. All data were obtained from inpatient settings. The follow-up endpoint was set as 24 months, which was the median of follow-up time.

### 2.3 Imaging Analysis

Contrast-enhanced CT scans were performed for all patients upon admission and during follow-up. Two senior radiologists independently performed the segmentation and measurement of the ascending aorta, following a standardized protocol throughout the process. The protocol followed was as follows: fixed monitoring parameters were established, and any controversial cases were referred to a third senior physician for arbitration. The measurers had access only to anonymous images and were blinded to clinical and follow-up information, ensuring that the data collected remained objective and reliable. Details regarding the acquisition protocol, diameter, and volume measurements have been documented previously [13]. The minimum slice thickness was set at 0.75 mm. The CTA datasets were analyzed using Horos® (Nimble Co LLC d/b/a Purview in Annapolis, MD, USA. 4 Version 3.3). The maximum and minimum diameters (mm) and aortic area (cm^2^) were measured on the double slopes near the aortic ring, aortic sinus, Sino-Tubular Junction (STJ), proximal ascending aorta, and the origin of the brachiocephalic trunk using 3D multi-plane reconstruction [12]. A centerline from the aortic annulus to the distal end of the descending aorta was created to assess the length between the annulus and the aortic arch. The volume measurements of the aortic root and ascending aorta were obtained through manual segmentation and subsequent creation of a 3D model to calculate the aortic volume automatically. The volume–height index (VHI) was computed by dividing the ascending aortic volume by the height of the patient, allowing for adjustment of height-related variations. In addition, the maximum aortic diameter and ascending aortic length were recorded.

### 2.4 Statistical Analysis

Continuous variables were presented as mean (standard deviation), and frequency and percentage were used to express the categorical variables. The chi-square test or Fisher’s exact test was employed for categorical variables, as deemed appropriate. The predictive value of the ascending aortic volume for adverse outcomes was evaluated using receiver operating characteristic (ROC) curve analysis, which included the determination of the optimal cutoff point. In addition, time-dependent ROC curve analysis was conducted utilizing the risk Regression package in R. A Cox proportional hazards model was developed with the ascending aortic volume being the primary covariate. To estimate the event-free survival, the Kaplan–Meier curves for the two groups were plotted, and the differences between the two groups were analyzed using the log-rank test. Cox regression analysis was conducted to investigate the role of ascending aortic volume in aortic progression, with adjustments made for age and sex. Statistical significance was set at a two-tailed *p*-value of <0.05. All statistical analyses were carried out using R software (version 4.1.3, R Foundation for Statistical Computing, Vienna, Austria).

## 3. Results

### 3.1 Baseline Characteristics

This study included a total of 101 patients with BAV. The mean age was 52 ± 22.65 years, with 65.35% of the patients being men. The average weight was 54.24 ± 20.14 kg, and the mean height was 1.53 ± 0.28 m. The mean ascending aortic volume was 99,496.51 mm^3^, and the average VHI was 62,242.28 mm^3^/m. Table 1 presents the other baseline characteristics.

**Table 1.** **Baseline characteristics of all study participants**.

	Data
Age (years)	52 ± 22.65
Male sex, n (%)	66 (65.35%)
Weight (kg)	54.24 ± 20.14
Height (m)	1.53 ± 0.28
Systolic blood pressure (mmHg)	118.15 ± 19.19
Diastolic blood pressure (mmHg)	66.09 ± 13.78
Hypertension, n (%)	22 (21.78%)
Diabetes mellitus, n (%)	3 (2.97%)
Cerebrovascular lesion, n (%)	5 (4.95%)
Chronic obstructive pulmonary disease, n (%)	0
Ascending aortic volume (mm^3^)	99,496.51 ± 59,142.90
Ascending aortic length (mm)	90.94 ± 22.34
Ascending aortic diameter (mm)	38.79 ± 10.40
Ascending aortic volume/height (mm^3^/m)	62,242.28 ± 34,640.99
Ascending aortic length/height (mm/m)	59.63 ± 11.63

### 3.2 Ascending Aortic Volume and Outcomes

**Fig. 1.**
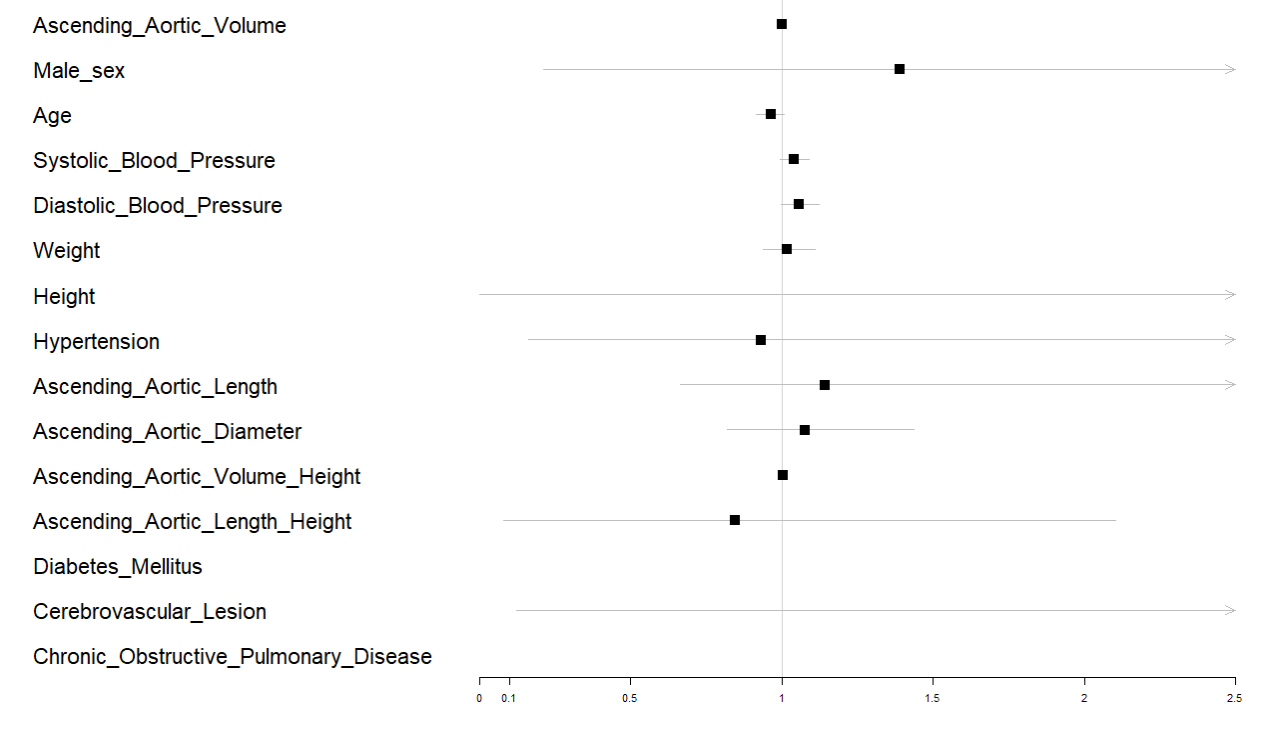
**The forest plot for the assessment of the association of demographic and clinical variables with adverse outcomes in patients with bicuspid aortic valve (BAV)**.

Twenty-four adverse events were observed, including 4 aortic-related deaths. The collected demographic and clinical variables were assessed using multivariable logistic regression analysis, and a forest plot (Fig. 1) was generated to determine their association with adverse outcomes in patients with BAV. There were no statistically significant differences for variables such as age (*p* = 0.118), sex (*p* = 0.734), weight (*p* = 0.686), height (*p* = 0.522), systolic blood pressure (*p* = 0.111), diastolic blood pressure (*p* = 0.076), hypertension (*p* = 0.931), diabetes (*p* = 0.991), cerebrovascular disease (*p* = 0.379), length of ascending aorta (*p* = 0.837), diameter of ascending aorta (*p* = 0.612), and ascending aortic length/height ratio (*p* = 0.874). The *p*-values for these variables were greater than 0.05. Only ascending aortic volume (*p* = 0.049) and VHI (*p* = 0.048) demonstrated statistically significant differences (Table 2).

**Table 2.** **Logistic regression analysis results for patients’ demographic and clinical variables**.

	OR value	*p* value	95% CI
Age (years)	0.9631	0.118	0.92–1.01
Male sex, n (%)	1.3880	0.734	0.21–10.00
Weight (kg)	1.0169	0.686	0.94–1.11
Height (m)	>10	0.522	
Systolic blood pressure (mmHg)	1.0372	0.111	0.99–1.09
Diastolic blood pressure (mmHg)	1.0547	0.076	0.99–1.12
Hypertension, n (%)	0.9292	0.931	0.16–4.76
Diabetes mellitus, n (%)	<0.1	0.991	
Cerebrovascular lesion, n (%)	4.3545	0.379	0.12–142.74
Ascending aortic volume (mm^3^)	0.9995	0.049*	0.99892–0.99987
Ascending aortic length (mm)	1.1398	0.837	0.66–4.59
Ascending aortic diameter (mm)	1.0735	0.612	0.82–1.44
Ascending aortic volume/height (mm^3^/m)	1.0008	0.048*	1.00023–1.00182
Ascending aortic length/height (mm/m)	0.8447	0.874	0.08–2.10

OR, odds ratio; CI, confidence interval. * indicates *p* < 0.05.

**Fig. 2.**
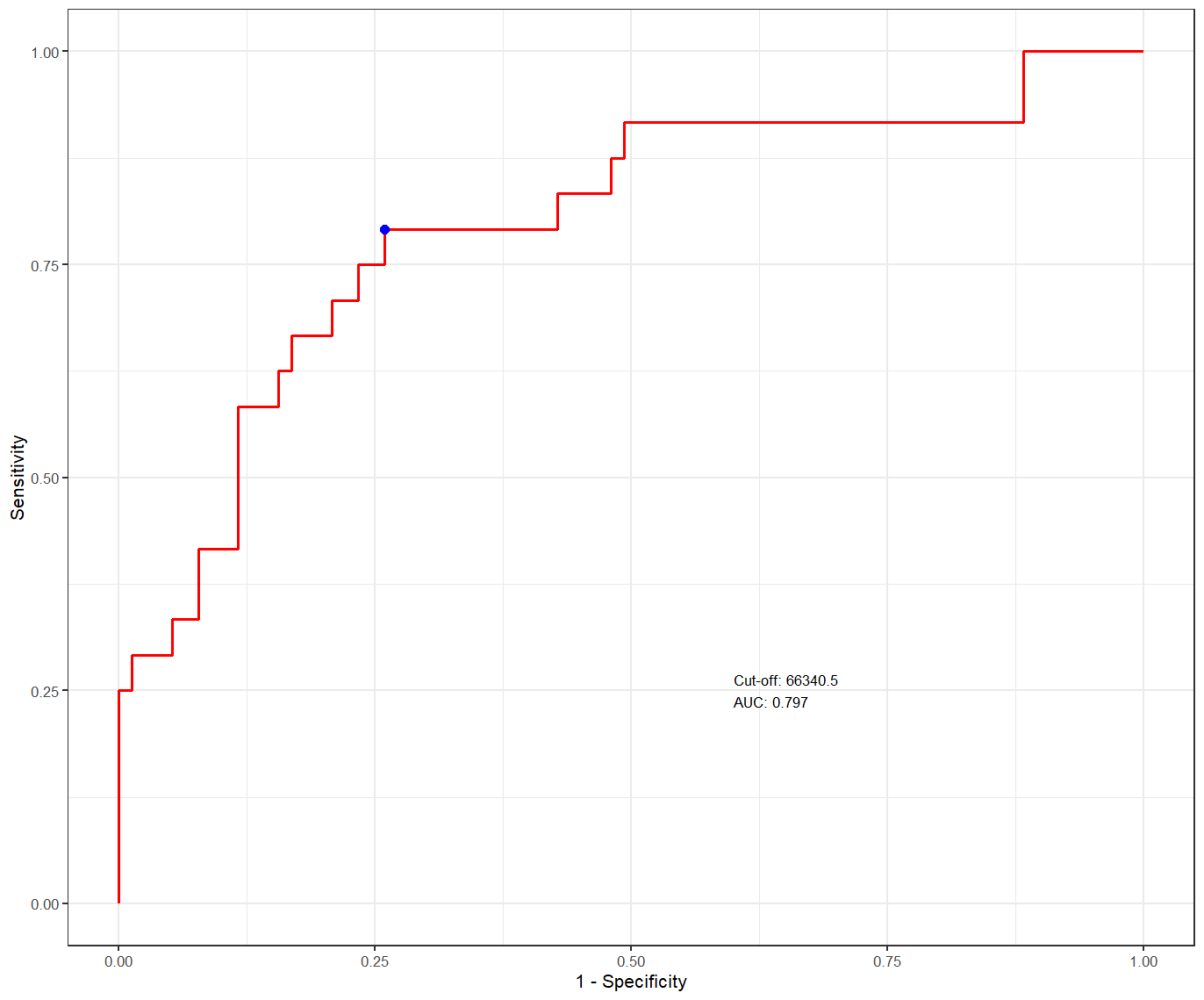
**The predictive value of volume–height index (VHI) for adverse outcomes was assessed using the receiver operating characteristic (ROC) curve analysis**.

**Fig. 3.**
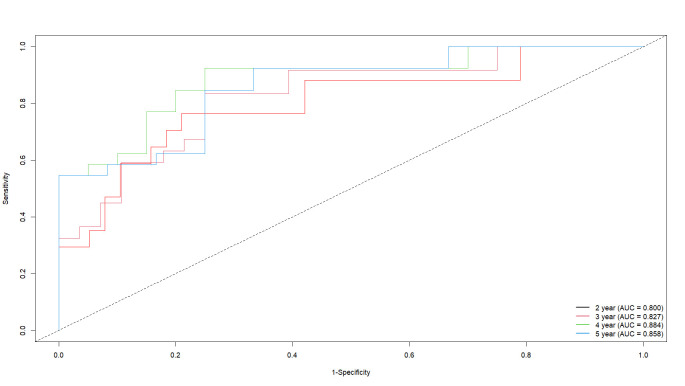
**The predictive value of volume–height index (VHI) for adverse outcomes was assessed using dynamic receiver operating characteristic (ROC) curve analysis**.

**Fig. 4.**
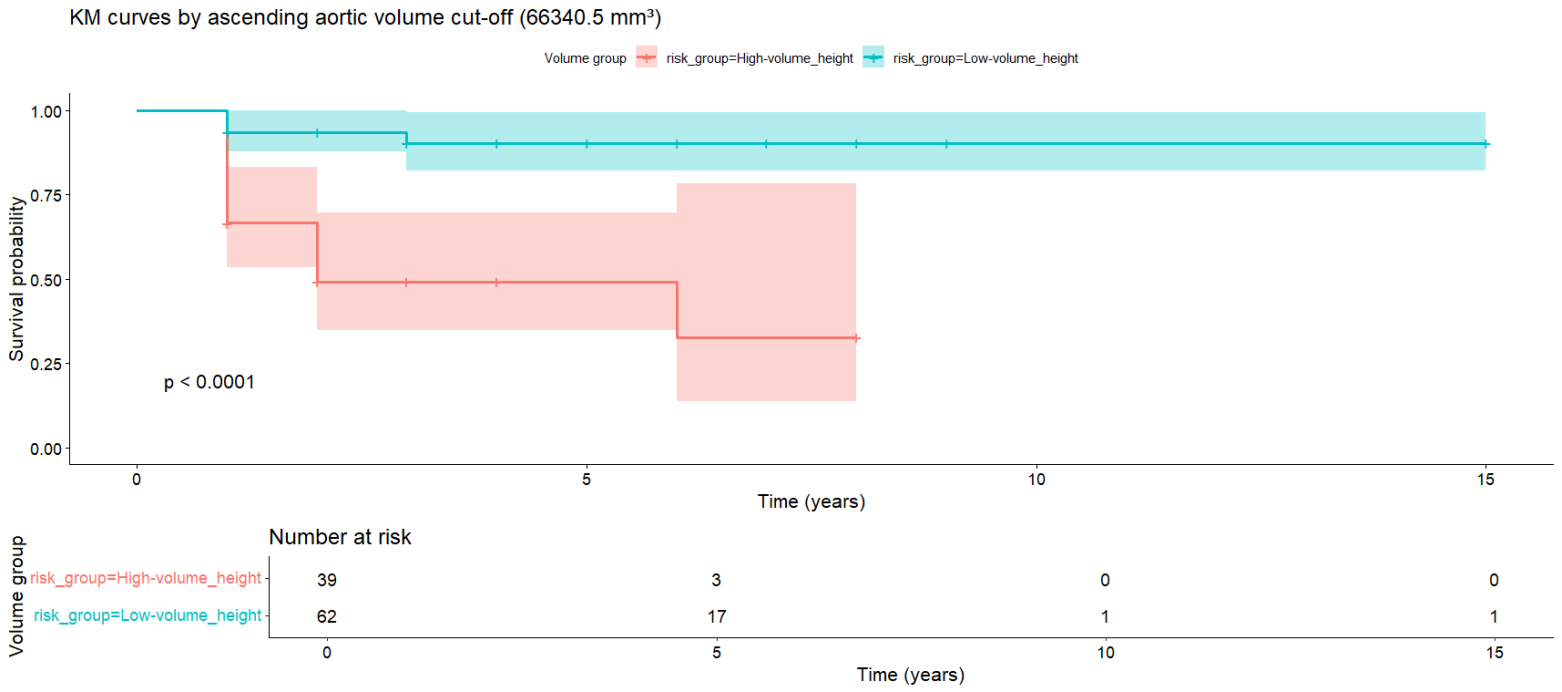
**Kaplan–Meier analysis for the adverse events during follow-up**.

The optimal VHI cutoff value was revealed as 66,340.5 mm^3^/m using ROC curve analysis, with an area under the curve (AUC) of 0.797 (95% confidence interval [CI], 0.676–0.896) (Fig. 2). The results of dynamic ROC curve analysis exhibit that the discriminatory ability of VHI in predicting aortic adverse events gradually improves in patients with BAV, with the extension of follow-up time. This reaches a peak at 3 to 4 years (AUC = 0.827–0.884), and maintains a high predictive ability even at the fifth year (Fig. 3). Patients were divided into a high-risk group (VHI ≥66,340.5 mm^3^/m) and a low-risk group (VHI <66,340.5 mm^3^/m) based on this cutoff value (Table 2). The event-free survival curves were plotted for the two groups using the Kaplan–Meier method (Fig. 4), and the log-rank test was used to compare the differences between the two groups. Kaplan–Meier analysis revealed that, based on the VHI cutoff value (66,340.5 mm^3^/m) determined by the ROC curve, when patients with BAV were divided into high-risk and low-risk groups, the cumulative event rate in the high-risk group was significantly higher within 15 years (*p* < 0.0001). This indicated that VHI can serve as an effective long-term predictive indicator, assisting in distinguishing high-risk individuals for aortic aneurysm, aortic dissection, and mortality. Based on this, VHI was further assessed by dividing the VHI range into quartiles, and the *p*-value was found to be less than 0.05 (*p* = 0.0392) when VHI ranged from 58,000 to 81,000 mm^3^/m.

## 4. Discussion

Traditionally, the decision to perform surgery in patients with BAV and aortic dilation has been based on the diameter of the ascending aorta [14]. This is because ascending aortic diameter is closely linked with genetic and hemodynamic factors, which help elucidate the development of aortic dilatation of aorta [15, 16].

The limitations of relying solely on aortic diameter have become clear over time. Many patients with aneurysms or aortic dissection do not fulfil the surgical threshold based on diameter, suggesting that this metric may not be universally predictive. This has resulted in a “leftward shift” in surgical indications, with surgical procedures being performed at smaller diameters. While this proactive approach facilitates earlier intervention, it may also lead to unnecessary surgeries for some patients, thereby increasing surgical risks without demonstrating clear benefits [17]. For example, this study revealed that almost all patients with an ascending aortic diameter exceeding 5.5 cm experienced adverse events within three years. In contrast, approximately half of those with a diameter greater than 4.5 cm experienced adverse events in the same period, while approximately 12% of patients with diameters less than 4.5 cm still had adverse outcomes. This discrepancy underscores the urgent need for additional indicators to enhance the existing surgical thresholds. A review of the literature suggests that, in addition to ascending aortic diameter, ascending aortic length plays a significant role in thoracic aortic aneurysms. This measurement is less influenced by dissection and may serve as a more reliable indicator. Aortic length can also be utilized as a criterion for intervention in thoracic aortic aneurysms and is considered more dependable than diameter in some cases [18]. Based on this, ascending aortic volume was selected for examination as a combined measure that accounts for both diameter and length. Our analysis demonstrated that ascending aortic volume was significantly associated with adverse outcomes in patients with BAV. Studies on aortic aneurysms have also indicated that linking aortic diameter with body height enhances prognostic value [19]. By employing this approach, the relationship between ascending aortic volume and patient height was explored, finding that the volume-to-height ratio was similarly associated with adverse outcomes. The findings of this study indicated that both the ascending aortic volume (*p* = 0.0338) and volume-to-height ratio (*p* = 0.0331) demonstrated a significant correlation with adverse outcomes. These findings suggested that ascending aortic volume is a strong predictor of adverse outcomes in patients with BAV. In addition, the volume-to-height ratio may serve as a supplementary or alternative measure to ascending aortic diameter for guiding surgical intervention decisions.

However, ascending aortic diameter in our multivariable analysis did not demonstrate predictive value. This may be because most patients in our study had an ascending aortic diameter less than 5.5 cm, which did not fulfill the surgical criteria defined by guidelines. Consequently, there was no statistically significant correlation between diameter and adverse events, highlighting its limitations. Therefore, the need for supplementary factors, such as ascending aortic volume and the volume-to-height ratio, is further supported to inform surgical decisions, particularly when diameter alone is not a sufficient indicator for intervention.

The volume of the ascending aorta can be regarded as an indicator for surgical intervention in patients with ascending aortic aneurysms, as it has a stronger correlation with pathogenic hemodynamic conditions. Meanwhile, measurements of ascending aortic volume indicate higher diagnostic accuracy for ATAAD compared to the maximum diameter, enabling timely identification of patients at risk. In our study, an increased likelihood of adverse events along with the ascending aortic volume and volume-to-height ratio was observed. From a pathological aspect, due to the altered valve morphology, abnormal flow patterns develop in the aorta with increased shearing of blood flow over the aortic wall, leading to aortic dilation. Patients with distinct baseline characteristics will develop aortic growth slowly but at different rates [20]. Additionally, diameter alone does not best identify patients at risk for aortic dissection, as aortic dissection typically occurs below the current aortic diameter threshold for aortic surgery. The combination of aortic diameters and aortic ascending length corrected for body height seems to be a better predictor of aortic dissection and rupture than diameter alone. In our study, the VHI was found to be independently associated with adverse outcomes, indicating that VHI is a sound predictor in this model. However, larger studies are required to confirm this finding and refine thresholds for these metrics.

Additionally, the logistic regression analysis results (Table 2) reveal that the odds ratio (OR) for ascending aortic volume is less than 1, whereas that for the ratio of ascending aortic volume to height is greater than 1. This discrepancy may be because height acts as a confounding variable in this study. Literature suggests a positive correlation between ascending aortic length and expansion of the aorta [21]. Consequently, there should also be a positive correlation between volume and adverse outcomes in this study. Therefore, it is appropriate to use the volume-to-height ratio as an indicator.

This study suggests that ascending aortic volume-to-height ratio, including both diameter and length, could serve as a valuable supplementary metric for the prediction of adverse outcomes in patients with BAV. Although ascending aortic diameter remains a critical factor, particularly when it exceeds established surgical thresholds in clinical decision-making, volume-based metrics may provide additional insights. This is particularly observed when the diameter alone is insufficient to indicate surgery. Owing to the limitations of the study, including its single-center design and relatively small sample size, these findings cannot be generalized to clinical practice. Surgical decisions should still adhere to guidelines based on the diameter of the aorta. However, incorporating the volume-to-height ratio into risk stratification for patients who do not meet surgical thresholds could assist in identifying those at higher risk and allow for earlier intervention, potentially improving the outcomes.

## 5. Limitations

This analysis is limited by its retrospective design, which may introduce selection bias. The data utilized for this study were obtained from the existing medical records, which might have contained inaccuracies, incompleteness, or inconsistencies, potentially leading to information bias. Additionally, the study is constrained by a small sample size and the absence of a control group. All data were collected from a single center, which may restrict the generalizability of our results. Moreover, ROC analysis was performed to identify the cutoff value to categorize the patients, which was subsequently used for Kaplan–Meier plotting. The authors aimed to investigate the correlation between VHI and adverse events; therefore, we did not assess whether the baseline characteristics were balanced between high- and low-risk groups. This potential imbalance in the baseline characteristics may result in bias within our findings. To address these limitations, future studies should emphasize high-quality clinical trials with larger sample sizes and extended follow-up periods to confirm these findings. Furthermore, specific clinical situations should be elucidated where treatment might need to be selected. Thus, conducting external validation with a larger sample size will also help to enhance the reliability of our findings.

## 6. Conclusion

Current treatment guidelines for BAV predominantly emphasize aortic diameter and its progression [12]. While surgical intervention thresholds can be flexible for certain patients, growing evidence indicates that relying solely on aortic diameter for the stratification of risk is insufficient. This study aims to identify additional predictive factors that complement existing guidelines, moving beyond size alone as a risk measure, ultimately improving surgical decision-making and enhancing the quality of life of patients.

## Availability of Data and Materials

The raw data of this study are kept in our institution and will be publicly available as of the date of publication. All data reported in this paper will also be shared by the lead contact upon request.
